# Case of Cerebral Inflammation

**Published:** 1823-09

**Authors:** T. Rolph

**Affiliations:** Crutched Friars.


### ART. V.—*Case of Cerebral Inflammation.* By T. ROLPH, Esq. Crutched Friars.

MISS TIDSWELL, aged twelve years, was taken, on the 6th of July, with slight pain in the head, which subsided and returned at intervals. On the 6th of July, the day I first saw her, she complained of a dull pain, with a sense of weight, but occasionally a very acute shooting pain across the forehead, accompanied with noise in the ears, giddiness, especially on stooping, throbbing of the temporal arteries, great heat across the forehead; the cheeks were alternately flushed and pale; the bowels were costive, the urine high-coloured; the tongue was furred, the skin hot and dry; she complained of thirst, had no appetite, and the pulse was 116. I inquired of her mother, and found that her sleep was very disturbed: she snatched a great deal in it, talked of things that occurred during the day, and frequently awoke much frightened from dreams. Her temper, which was generally excellent, became peevish and irritable. With these symptoms, I judged it prudent to abstract blood, and accordingly applied three cupping-glasses, and took away eight ounces of blood. I gave her Hyd. Submuriat. gr. x.; Ext. Rhæi, ℈ij. divide in pil. xij. sumat duo nocte maneque. —℞. Magnes. Sulph. ℥j.; Sp. Mindereri, f. ℥j.; Mist. Camph. f. ℥vij. ft. Mist. sumat coch. iii. 6ta quaque hora.

I called again to see her on the 8th, and was sorry to find no amendment, but rather that the pain in the head increased. I proposed bleeding the child again; but, the parents not appearing very willing, I had the head shaved, a blister applied to the nape of the neck; ordered the aperient mixture to be continued, but, in addition, I gave her Pulv. Jalapæ, gr. x.; Hyd. Sub. gr. iv. which I ordered to be taken immediately, and the blister to be kept open with the sabine ointment.

I called again on the 10th, and found her evidently better; the tongue cleaner, the head easier, the bowels more open, the skin moist, the pulse 98; and heard that she had rested better.

When I next saw her, which was on the 12th, all the symptoms had returned with increased violence. I gave her another purgative powder; directed that the following cold lotion should be kept constantly applied to the head,—viz. ℞. Ammon. Mur. Potassæ Nitrat., āā ℥j.; Acet. distillat. ʒvj.; Aquæ font. ℔j. M. ft. lotio assidué applicanda; and the feet should be kept in warm water for twelve minutes every hour. I also urged very strongly the necessity of confining her to lemonade and a little fruit, as I was fearful they had rather deviated from what I had enforced in that respect.

On the 13th I saw her. Her pulse was 120; she appeared then, for the first time, to have an aversion to the light; her bowels were more inactive, and her head in more pain. I gave her the same purgative, which, as before, appeared to relieve her.

On the 14th I saw her again, and found her worse. I proposed bleeding again, but her parents, as before, appeared very averse to it. I recommended them to call in Dr. Birkbeck, who saw the child with me the same evening, and immediately coincided in the propriety of blood-letting. Her pulse was 130 to 140; her forehead felt extremely hot, her tongue was very much furred, her head was in very acute pain; light was very painful to her, and she complained of pain in her eyes; her skin was hot, her feet cold, her cheeks very much flushed, and her thirst extreme. I took from her arm, in a full stream, ten ounces of blood; and Dr. B. ordered the following medicine: viz.—℞. Magn. Sulphat. ʒj.; Inf. Sennæ, ℥iij.; Tinct. Digit. m. x.; Syr. simpl. ʒj.; Aq. font. ℥vj. fiat haustus sextâ quâque horâ sumendus. The blood was buffy.

On the 15th she was much better; had passed a more comfortable night; her sleep was less disturbed; her pulse was 110; her skin was cooler, and her head very much relieved. Persistat in usu haustus heri prescripti.

16th.—Much the same; her bowels had been opened this day seven times.

17th.—Pulse 112; bowels open five times; fever abated, thirst less, tongue cleaner, skin moist, although the head was rather worse. Ordered the cold lotion to be applied assiduously, the room to be kept cool, and the child quiet; also the following draughts to be taken, leaving out the Infus. Sennæ, as the bowels had been so copiously opened:—℞. Magnesiæ Sulph. ʒj.; Tinct. Digitalis, m. x.; Sp. Æther. Nit. m. xx.; Syrupis Aurantii, ʒj.; Aquæ fontanæ, ℥vij. fiat haustus sextâ quâque horâ sumendus.

18th.—Pulse 100; bowels open; head free from pain, and other marks of general amendment. I had told her the day before, whenever the head was in pain or the face hot, to bathe the forehead and temples well with the cold lotion: this she had done, and the relief that followed she assured me was extraordinary. I directed the draughts to be continued.

19th.—The amendment from yesterday was striking; the pulse had fallen to 90. This being the case, I lowered the tincture of digitalis to m. v. each draught, and the sulphate of magnesia to ℈ij. I gave permission to eat a little bread-pudding, and to take a little milk. From this time Dr. Birkbeck discontinued his visits; the amendment was gradual and sure, the child being at this time in good health.

I am convinced that, had not this disease been arrested in its commencement, it would have ended in hydrocephalus: the symptoms were strongly marked; the child was evidently of a strumous habit; the skin extremely thin, smooth, and fair; the hair light, and very fine; large blue eyes, thick upper lip, prominent veins, and laxity of muscular fibre; her intellectual powers were very lively and acute. This is the habit in which hydrocephalus is likely to be formed, and these were the symptoms which in general precede it. I have long felt assured that the only time to attack hydrocephalus successfully is at its onset: even then the recovery is doubtful; in the second stage, the chance is much lessened, and the prognosis must be very unfavourable; but, in the third, it is uniformly hopeless. To combat this disease, recourse must be had to the most powerful remedies as early as possible, and these must be maintained with vigour. Bleeding is without doubt the most powerful agent we can employ, and this must be carried as far as possible consistent with the safety of the child. Another main remedy is purging freely, and giving the digitalis till it lowers the pulse; but the striking advantage resulting from having the head shaved, and the coldest applications kept constantly applied to it, must not be overlooked. Blisters also are extremely useful. I conceive the indication of cure consists in keeping, as much as possible, the blood from the brain; in keeping the system quiet by total abstinence from food; and reducing the inflammation, by keeping the patient cool, quiet, and following most rigorously the antiphlogistic treatment. Both when I cupped, as well as when I bled her, I did it till she fainted, and was much gratified to find how efficacious it proved; and am sure that where bleeding fails of doing good, in cases of severe cerebral excitement, it is not the fault of the remedy, but the inefficient manner in which it had been employed.

*July 25th,* 1823.

